# Gram-negative bloodstream infections: where can we do better? A retrospective cohort study

**DOI:** 10.1007/s10096-025-05231-4

**Published:** 2025-08-09

**Authors:** Manouc Guit, Konstantin Tanida, Nicole Degel-Brossmann, Martin Christner, Martin Aepfelbacher, Holger Rohde, Flaminia Olearo

**Affiliations:** 1 Center for Diagnostics, Institute of Medical Microbiology, Virology and Hygiene, University Medical Center Hamburg-Eppendorf, Hamburg, Germany; 2 Antimicrobial Stewardship Team, University Medical Center Hamburg-Eppendorf, Hamburg, Germany

**Keywords:** Gram-negative bloodstream infection (GN-BSI), Antimicrobial stewardship (AMS), Antibiotic de-escalation (ADE), Oralization, Uncomplicated GN-BSI

## Abstract

**Purpose:**

Gram-negative bloodstream infections (GN-BSI) significantly impact hospital admissions, presenting major health challenges. Despite guidelines advocating de-escalation, oralization, and appropriate treatment durations, real-world clinical management remains unclear.

**Methods:**

This retrospective observational study assessed GN-BSI management at a tertiary care hospital, comparing uncomplicated (uGN-BSI) and complicated (cGN-BSI) cases from January to December 2022. It focused on identifying risk factors for suboptimal therapy, defined as failure to adopt the narrowest effective spectrum suggested by susceptibility reports within 24 h of result availability.

**Results:**

Among 194 patients studied, 52.1% had uGN-BSI which were predominantly caused by *Escherichia coli* (54.6%) with a urinary tract source, while cGN-BSI showed higher rates of AmpC producers (22.6%) and *Pseudomonas aeruginosa* (8.6%). Treatment durations deviated by a median of + 2 days (interquartile 0–5) for cGN-BSI. Missed opportunities for oralization were higher in uGN-BSI (76.2%) than in cGN-BSI (55.9%). Average time to oralization was 5.5 days in uGN-BSI versus 6.5 days in cGN-BSI. Rates of optimal treatment initiation within 24 h post-antibiogram were low (uGN-BSI: 22.8%, cGN-BSI: 26.9%). Third-generation cephalosporine resistant isolates (OR 0.3, CI95% 0.1–0.9) and AmpC-producers (OR 0.3, CI95% 0.1–0.8) were least associated with suboptimal therapy, while urinary tract sources in uGN-BSI trended to pose higher risk. cGN-BSI patients had fewer missed oralization opportunities than uGN-BSI patients, with a protective trend in the multivariate (OR 0.5, CI95% 0.2-1).

**Conclusion:**

GN-BSI management frequently does not meet guideline standards, especially in de-escalation and oralization. uGN-BSI could benefit from antibiotic stewardship interventions, whereas cGN-BSI requires tailored strategies, including individualized ID consultations.

**Supplementary Information:**

The online version contains supplementary material available at 10.1007/s10096-025-05231-4.

## Introduction

Gram-negative bloodstream infections (GN-BSI) represent a leading cause of hospital admission among the community-acquired infections and are associated with significant morbidity and mortality [1, 2]. These infections necessitate prompt and effective antimicrobial therapy and rigorous source control measures to mitigate their life-threatening consequences [3, 4]. Evidence highlights the critical role of rapid diagnostic tests and the presence of antimicrobial stewardship teams in enhancing outcomes for patients with bloodstream infections [5, 6]. Additionally, evolving strategies in the management of GN-BSI, such as reducing treatment durations, de-escalating antimicrobial therapy, and early transition from intravenous to oral therapy, have gained attention. Recent randomized controlled trials (RCTs) have demonstrated the non-inferiority of early oralization as well as shorter treatment periods of 7 instead of 14 days of antibiotic therapy [7–12]. Similarly, de-escalation has been proven to be safe and to prevent the emergence of multidrug resistant bacteria [13, 14]. However, these trials predominantly involve patients with uncomplicated bacteremia, raising questions about the applicability of these findings to more complex cases (such as those requiring prolonged treatment or with no possible source control) or immunocompromised individuals [15]. In fact, complicated GN-BSI (cGN-BSI) represent a heterogeneous group with various risk factors for the development of complications or the occurrence of treatment failure [16, 17]. While available guidelines are of limited applicability to patients with complicated GN-BSI, real world data on the actual management of these cases is also scarce. This study aims to evaluate the management of GN-BSI at a tertiary care university hospital in Germany, where rapid diagnostic testing, AMS, and consultation with infectious disease (ID) specialists are routinely available and a special focus will be placed on differences in the management approaches between uncomplicated and complicated GN-BSI cases. Additionally, we try to identify risk factors for suboptimal therapy, including missed opportunities for de-escalation or escalation, transition to oral therapy, and inappropriate duration of antimicrobial treatment.

## Methods

### Study design

This retrospective observational study was conducted at the University Medical Center Hamburg-Eppendorf, Germany a tertiary-care university hospital with approximately 1700 beds. The study covered a one-year period from January to December 2022.

### Inclusion and exclusion criteria

All patients aged > 18 years with positive blood cultures for *Enterobacterales* or *Pseudomonas aeruginosa* who did not have positive blood-cultures with other bacteria in the 10 days preceding and 10 days following the *Enterobacterales* bacteremia were screened for eligibility. Patients were excluded from the study under any of the following conditions: (1) those who underwent hematopoietic stem cell transplantation, (2) those who received a solid organ transplant within the first-year post-transplantation, (3) patients with incomplete or ambiguous medical record (4) those who were transferred or discharged from the hospital prematurely; (5) patients who died within the first five days after their initial positive blood culture.

## Definitions

We categorized GN-BSI as either complicated or uncomplicated [15]. Following criteria had to be fulfilled to be considered as uncomplicated: absence of immunosuppression, source of infection identified and execution of adequate source control, clinical improvement or at least stabilization within the first 72 h with afebrility and hemodynamic stability. Causative *Enterobacterales* were categorized as *Escherichia coli*,* Klebsiella spp.* (*Klebsiella pneumoniae*, *Klebsiella oxytoca*) or AmpC producers (*Citrobacter freundii*, *Enterobacter cloacae complex*, *Klebsiella aerogenes*, *Serratia marcescens*).

## Outcomes

This study aimed to identify risk factors for suboptimal treatment. Primary outcome was the missed opportunity to administer the most appropriate antibiotic, based on the narrowest effective spectrum indicated by the antibiogram, within the first 24 h of the antibiogram results becoming available. Species identification and antibiotic susceptibility testing were performed using the automated VITEK^®^ 2 system, following EUCAST breakpoint guidelines. Secondary outcomes included suboptimal oralization, focusing on the timing and selection of antibiotics for the transition from intravenous to oral therapy within 96 h of a positive GN-BSI result in patients who meet the criteria for oralization and inadequate duration of antimicrobial therapy, defined as a treatment duration that deviates by at least 72 h (either shorter or longer) from the standard duration recommended by international and local ID guidelines.

Further exploratory outcomes, not included in the primary risk factor analysis, assessed appropriateness and accuracy of antibiotic dosing, especially in the management of *P. aeruginosa* infections as well as adequacy of empirical treatment, reviewing the effectiveness of initial empirical antibiotic therapy in accordance with subsequent antibiogram results.

### Data collection

Data for this study were obtained from the electronic health record of our hospital. Recorded data included demographics, clinical and laboratory information related to the infection, Charlson-Comorbidity-Index (CCI), type of implanted foreign material, length of stay, source of infection, 90-day hospital readmission, 90-day all-cause mortality, and 90-day bacteremia recurrence. Immunocompromised status was collected, defined by one or more of the following: neutropenia with an ANC < 500 cells/mL, CD4 cell count < 200 cells/mL, corticosteroids > 20 mg of prednisolone equivalent per day over 14 days or cumulative > 700 mg, or immunomodulator therapy. Information on source control was also collected, defined as the execution of necessary interventions to manage the infection effectively. This includes the removal of infected foreign bodies, drainage of purulent fluids or abscesses, execution of interventions that were considered mandatory for source control or imaging diagnostic to rule out the presence of any complicating factors.

### Statistical analysis

Categorical variables were analyzed by Chi2 test or Fisher’s exact test, as appropriate, and continuous variables were analyzed by Student’s t-test or the Mann–Whitney test, depending on data distribution. A two-sided P value < 0.05 was considered statistically significant for all tests.

Logistic regression was used to evaluate risk factors associated with the primary and secondary outcomes (suboptimal antimicrobial treatment, missed opportunities for oralization, and inadequate treatment duration). The main independent variable was the classification of GN-BSI as uncomplicated (uGN-BSI) or complicated (cGN-BSI), with age and sex included as a priori covariates. Potential confounders were assessed for association with both the outcome and primary independent variable in univariate analyses and were incorporated into the multivariable model using a stepwise approach, based on their impact on model fit and potential to reduce confounding.

Statistical analysis was performed using STATA 16.0 (StataCorp, College Station, TX, USA).

## Results

**Fig. 1:**
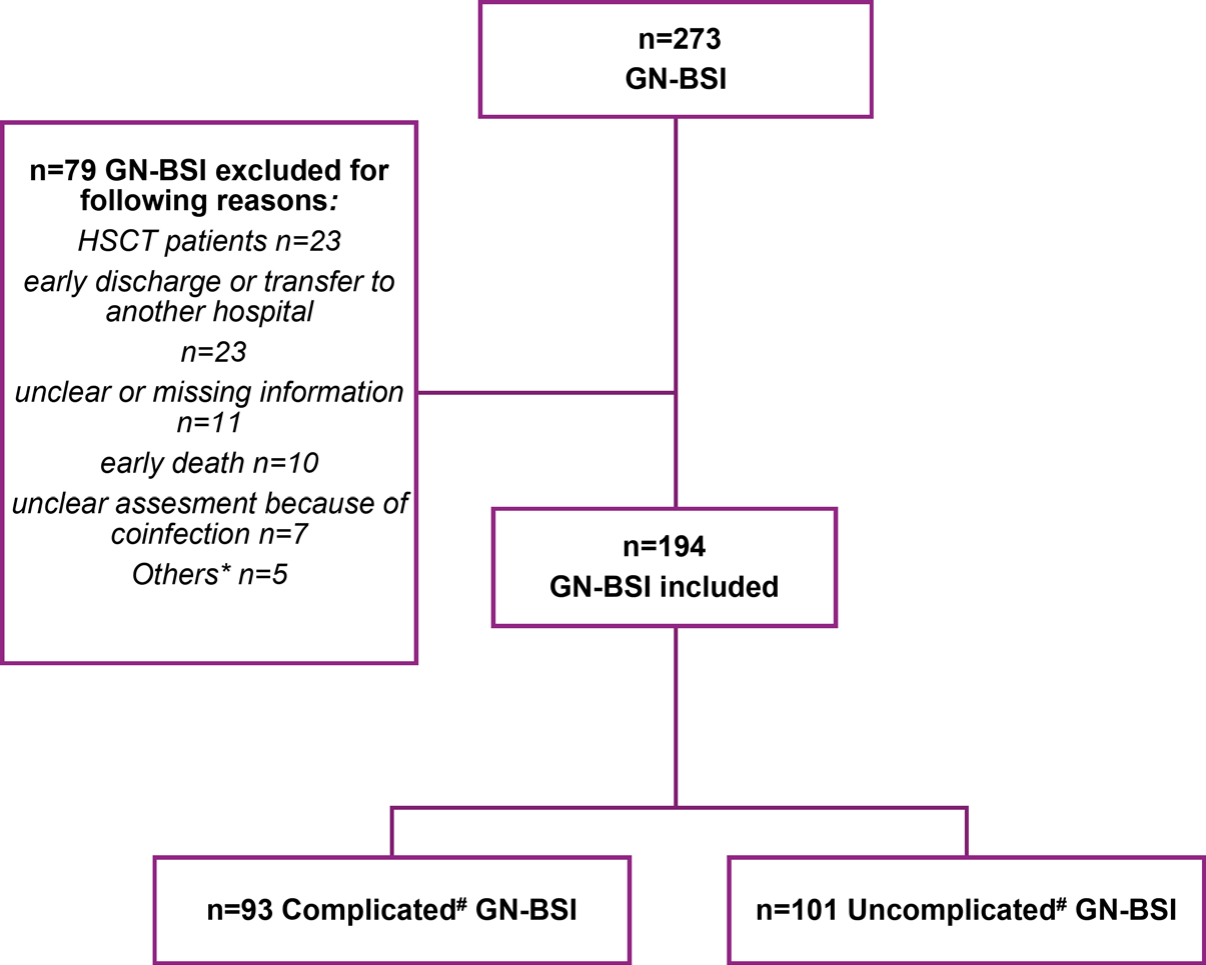
Flow chart, showing the inclusion of all screened patients in the complicated or uncomplicated group. GN-BSI: gram-negative bloodstream infection. Bloodstream infection was classified as either complicated or uncomplicated based on the Delphi criteria [1]. *Others: solid organ transplantation within first year post-transplant (*n* = 1), no admission to hospital (*n* = 4)

A total of 273 patients with positive blood cultures for *Enterobacterales* (*Escherichia coli*,* Klebsiella spp.*,* Citrobacter freundii*, *Enterobacter cloacae complex*, *Klebsiella aerogenes*, *Serratia marcescens)* or *P. aeruginosa* were assessed for eligibility. Of these, 79 were excluded based on predefined criteria (Fig. 1). Consequently, 194 patients with GN-BSI were included in the analysis. Out of the included cases, 52.1% (101/194) were classified as uGN-BSI. The remaining 93 cases were categorized as cGN-BSI due to one or more of the following factors: immunosuppression 53.8% (50/93, unidentified infection source 29% (27/93), or unsatisfactory clinical improvement within the first 72 h 41.9% (39/93).

### Baseline characteristics

The mean age was 64 years (SD 16.7), 48.5% of patients (94/194) were female and mean Charlson Comorbidity Index (CCI) was 5 (IQR 3–6). Patients with uGN-BSI were typically older, with a mean age of 69.2 years (SD 16.8), compared to 58 years (SD 14.8) for those with cGN-BSI. *E. coli* was more prevalent as the etiological pathogen in uGN-BSI, accounting for 54.6% (55/101) of cases, versus 45.2% (42/93) in cGN-BSI. In contrast, cGN-BSI cases had a higher prevalence of AmpC producers and *P. aeruginosa*, with 22.6% (21/93) and 8.6% (8/93) respectively, compared to 8% (8/101) and 6.9% (7/101) in uGN-BSI (p-value < 0.01, Table 1). Resistance to third-generation cephalosporins (3GCephRE) was observed in 14.4% of cases (28/194), while carbapenem resistance was detected in only two isolates (VIM and one NDM carbapenemase); however, no statistically significant difference in the distribution of resistance patterns was found between uGN-BSI and cGN-BSI (p value 0.52 and 0.17, respectively). Although the presence and the type of implanted foreign material did not differ significantly between complicated (19/93, 20.4%) and uncomplicated GN-BSI (19/101, 18.8%, *p* = 0.16), central lines and ports were present more often in cGN-BSI (30/93, 32.3% vs. 13/101, 12.9% in uGN-BSI, *p* < 0.01). The sampling of blood cultures from the emergency department vs. blood cultures taken by inpatient was more common for uGN-BSI (56.7%, 57/101) compared to cGN-BSI (38.7%, 36/93, *p* = 0.01). The most common source for GN-BSI was the urinary tract (UTI) with 97/194 cases (50%), followed by abdominal infections 34/194 (17.5%), endovascular device or catheter infections 13/194 (6.7%), pulmonary infections 12/194 (6.2%) and wound infections 7/194 (3.6%). In 27/194 (29%) of cases the source of infection was unknown. UTIs were much more common in uGN-BSI (66.3%, 67/101 vs. 32.2%, 30/93). On the other side, pulmonary infections (8/93, 8.6% vs. 4/101, 4%) and endovascular device or catheter infections (7/93, 7.5% vs. 6/101, 5.9%) were more common in cGN-BSI. ID consultation occurred in just 25/194 cases (12.9%). Although the frequency of ID consultations did not significantly differ between uncomplicated and complicated GN-BSI, the time to consultation was substantially longer for cGN-BSI, with a median of 6.5 days (IQR 2.5–11.5) compared to 2 days (IQR 1–2) for uGN-BSI (p- value = 0.01).

**Table 1:** Baseline characteristics overview for GN-BSI, stratified by uncomplicated and complicated cases

	Overall GN-BSI (*n* = 194)	Uncomplicated (*n* = 101)	Complicated (*n* = 93)	Comparison between complicated and uncomplicated GN-BSI (*p*-value)
Age (years), mean (SD)	64 (16.7)	69.2 (16.8)	58 (14.8)	**< 0.01**
Female, n (%)	94 (48.5)	53 (52.5)	41 (44.1)	0.24
Bacteria, n(%)	*E.coli*, 97 (50) Klebsiella spp*, 42 (21.6) AMPc producer, 29 (15) *P.aeruginosa*, 15 (7.7) *P.mirabilis*, 11 (5.7)	*E.coli*, 55 (54.6) Klebsiella spp*, 21 (20.9) AMPc producer*, 8 (8) *P.aeruginosa*, 7(6.9) *P.mirabilis*, 10 (9.6)	E.coli, 42 (45.2) Klebsiella spp*, 21 (22.6) AMPc producer*, 21 (22.6) *P.aeruginosa*, 8 (8.6) *P.mirabilis*, 1 (1)	**< 0.01**
Multiple-Drug resistant bacteria, n (%)	- Third Gen. Cephalosporine resistant (3GCephRE) 28 (14.4): - Confirmed ESBL 15 (7.7), - Carbapenem resistant 2 (1): - CARB-B VIM qPCR - CARB-B NDM qPCR	- Third Gen. Cephalosporine resistant (3GCephRE) 13 (12.9): - Confirmed ESBL 9 (8.9)	- Third Gen. Cephalosporine resistant (3GCephRE) 15 (16.1): - Confirmed ESBL 6 (6.5) - Carbapenem resistant 2 (2.2): - CARB-B VIM qPCR - CARB-B NDM qPCR	0.52 0.17
Number of blood culture pairs positive, median day (IQR)	1(1–2)	1(1–2)	1(1–2)	0.21
Number of positive blood culture days, median day (IQR)	1(1–1)	1(1–1)	1(1–1)	0.17
Charlson-Comorbidity-Index, median (IQR)	5 (3–6)	5 (3–6)	4 (3–6)	0.49
Implants	*n* = 38 (19.6%) • prosthetic joints 11 (5.7%) • implanted urinary tract devices 11 (5.7%) • endovascular devices 3 (1.5%) • vascular grafts 11 (5.7%) • artificial heart valves 2 (1%)	*n* = 19 (18,8%) • prosthetic joints 8 (7.9%), • implanted urinary tract devices 4 (4%), • endovascular devices 3 (3%) • vascular grafts 2 (2%) • artificial heart valves 2 (2%)	*n* = 19(20.4%) • prosthetic joints 3 (3.2%), • implanted urinary tract devices 7 (7.5%) • vascular grafts 9 (9.7%)	0.16
Central venous catheter, Port, Dialysis-central Line	*n* = 43 (22.2)	*n* = 13 (12.9%)	*n* = 30 (32.3%)	**< 0.01**
Blood-culture from emergency room vs. ward, n (%)	93 (47.9)	*n* = 57 (56.4%)	36 (38.7)	**0.01**
Source of infection, n (%)	Unknown, 27 (13.9) Urinary tract infections, 97 (50) Abdominal infections, 34 (17.5) Pulmonary infections, 12 (6.2) Endovascular device or catheter infection, 13 (6.7) Wound infections, 7 (3.6)	Urinary tract infections, 67 (66.3) Abdominal infections, 19 (18.8) Pulmonary infections, 4 (4) Endovascular device or catheter infection, 6 (5.9) Wound infections, 4 (4)	Unknown, 27 (29) Urinary tract infections, 30 (32.2) Abdominal infections, 15 (16.1) Pulmonary infections, 8 (8.6) Endovascular device or catheter infection, 7 (7.5) Wound infections 3 (3.2)	**< 0.01**
Infectious disease consultation, n (%)	25 (12.9)	13 (12.9)	12 (12.9)	0.9
Time to Infectious disease consultation, day (IQR)	2 (1–5)	2 (1–2)	6.5 (2.5–11.5)	**0.01**

*except *K. aerogenes*

*Citrobacter spp,* Enterobacter cloacae complex*,* K.aerogenes*,* Serratia marcescens*

### Antimicrobial therapy characteristics

**Fig. 2:**
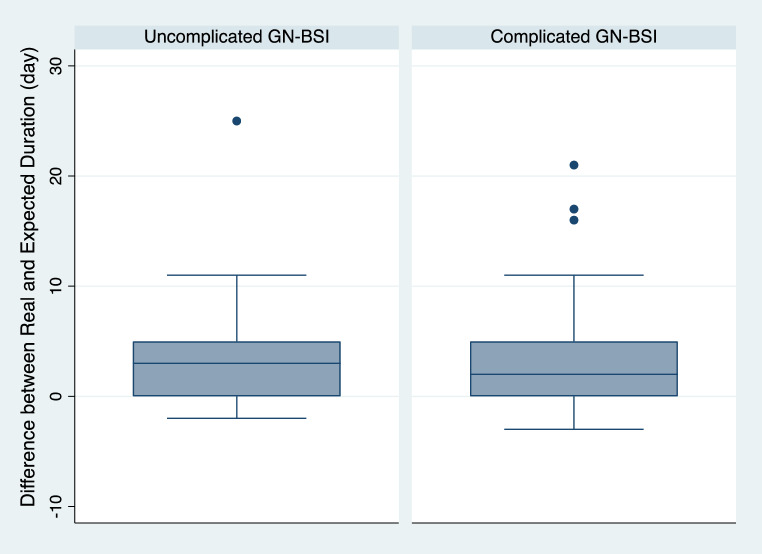
Box plot for Comparison between difference among real and expected duration in antimicrobial therapy by uncomplicated and complicated GN-BSI (p value 0.49)

The median antibiotic duration was longer in cGN-BSI (12 days, IQR 9–15 days, vs. 10 days, IQR 7–13, *p* < 0.01). There was a median deviation from the expected duration of + 2 days (range − 3 to + 25 days, Fig. 2), with no statistically significant difference observed between uGN-BSI and cGN-BSI (p-value = 0.49). The rate of oralization was higher in uGN-BSI at 43% (40/93) compared to 31.1% (23/74) in cGN-BSI (p- value 0.11), with a median time to oralization overall of 6 days (IQR 4–8 days, Table S1). The most frequently used oral antibiotics for GN-BSI were Ciprofloxacin (26/63, 44.4%) and Amox/Clav (17/63, 27%). TMP/SMX was used in only 5/63 cases (7.9%, Table S1).

### Treatment outcomes

The overall rate of optimal treatment within the first 24 h after availability of the antibiogram was 24.7% (48/194), with no significant differences observed between uGN-BSI and cGN-BSI (22.8%, 23/101 versus 26.9%, 25/93, p-value 0.51, Table 2). Delayed optimal treatment, occurring more than 24 h after the antibiogram, was also achieved in 24.2% of cases (47/194). It was more common in uGN-BSI (27.7%, 28/101 vs. 20.4%, 19/93), though not statistically significant (*p* = 0.24). The median time to delayed optimal treatment was shorter in uGN-BSI (3 days vs. 4 days, *p* = 0.12, Table 2, Figure S1). Missed opportunities for oralization were more common in uGN-BSI (76.2%, 77/101 vs. 55.9%,52/93 *p* < 0.01, Figure S2). The length of hospital stay after positive blood cultures averaged 9 days (IQR 5–16), significantly shorter for uGN-BSI (7 days [IQR 5–11] vs. 13 days [IQR 7–23], *p* < 0.01). 90-day all-cause mortality was 13.9% (27/194), substantially higher in cGN-BSI (23.7% vs. 4.9%, p-value < 0.01). Complicated cases also had higher rates of 90-day hospital readmission (47.3% vs. 31.7%, p-value 0.01). Although not statistically significant, cGN-BSI was associated with a higher rate of 90-day bacteremia recurrence compared to uGN-BSI (7.5% vs. 3%, *p* = 0.15; Table 2). Notably, recurrence occurred in only 1 of 63 patients who received oral step-down therapy, compared to 9 of 130 patients who did not receive any oral treatment (*p* = 0.118). Our study found no significant differences in the risk of suboptimal therapy between uGN-BSI and cGN-BSI (cGN-BSI OR 1.1, 95% CI 0.5–2.3, *p* = 0.76, Table 3). 3GCephRE was associated with a decreased likelihood of suboptimal therapy (OR 0.3, 95% CI 0.1–0.8, p-value < 0.01). Similarly, AmpC-producing bacteria also showed a protective effect, significantly reducing the risk of suboptimal treatment (OR 0.3, 95% CI 0.1–0.9, *p* = 0.03). Patients with cGN-BSI were less likely to experience missed opportunities for oralization compared to those with uGN-BSI, showing a significant association in univariate analysis (OR 0.4, *p* < 0.01) that approached significance in the multivariate model (OR 0.5, *p* = 0.07, Table S2). Interestingly, among the sources of infection, only abdominal sources showed a marginally increased risk in the univariate model for missed opportunity of oralization (OR 2.3, *p* = 0.05), though this was not confirmed in the multivariate analysis.

**Table 2:** Outcomes stratified by uncomplicated and complicated GN-BSI

Variable	All (*n* = 194)	Uncomplicated (*n* = 101)	Complicated (*n* = 93)	Comparison between complicated and uncomplicated GN-BSI (*p*-value)
Optimal Treatment (OT)*, n (%) <=24 h since the available antibiogram	48 (24.7)	23 (22.8)	25 (26.9)	0.51
Optimal Treatment (OT) lately achieved (> 24 h after antibiogram), n (%)	47 (24.7)	28 (27.7)	19 (20.4)	0.24
Time to optimal Treatment (when delayed), median day (IQR)	3 (3–5)	3 (2.5-4)	4 (3–6)	0.12
Length of Stay, median day (IQR)	9 (5–16)	7 (5–11)	13 (7–23)	< 0.01
All-cause Mortality 90d	27 (13.9)	5 (4.9)	22 (23.7)	< 0.01
90 d GN-BSI recurrence	10 (5.1)	3 (3)	7 (7.5)	0.15
Hospital Readmission 90 d, n (%)	76 (39.2)	32 (31.7)	44 (47.3)	0.02
Inadequate empirical Treatment	26 (13.4)	13 (12.9)	13 (13.9)	0.82

*Optimal therapy is defined as the direct opposite of suboptimal therapy

**Table 3:** Risk factors for suboptimal therapy in univariate and multivariate model (*N* = 194)

		Univariate Odds-Ratio	*P* value	Multivariate Odds-Ratio	*P* value	
Uncomplicated BSI Complicated BSI		1 0.8 (0.4–1.5)	0.5	1 1.1 (0.5–2.3)	0.76	
Age	<=64 y-o > 65	1 1.1 (0.6–2.1)	0.74	1 1.2 (0.6–2.6)	0.57	
Sex	Female Male	1 0.9 (0.5–1.7)	0.67	1 1.09 (0.5–2.3)	0.82	
Bacteria	*E. Coli* AmpC *Klebsiella spp.* *P. mirabilis* *P. aeruginosa*	1 0.3 (0.1–0.7) 1 (0.4–2.4) 2.8 (0.3–22.8) 1 (0.3–4.3)	**< 0.01** 0.97 0.35 0.88	1 0.3 (0.1–0.9) 1 (0.4–2.6) 2.4 (0.3–20.7) 0.9 (0.2–3.6)	**0.03** 0.94 0.42 0.87	
Third generation cephalosporin resistant (3GCephRE)	Not resistant Resistant	1 0.3 (0.1–0.6)	**< 0.01**	1 0.3 (0.1–0.8)	**< 0.01**	
Source	Urinary Unknown Abdominal Pulmonary Endovascular device Wound Other	1 0.8 (0.3–2.5) 1.8 (0.7–4.5) 2.1 (0.4–11.3) 0.6 (0.2–2.5) 1 (0.2–6.3) 0.4 (0.1–3.4)	0.74 0.19 0.39 0.94 0.96 0.4			
Immunosuppression	No Yes	1 0.7 (0.3–1.4)	0.3			
Charlson index	> 5 3–4 1–2 0	1 1(0.5–2.4) 1.2 (0.4–3.3) 1.1 (0.3–3.7)	0.8 0.7 0.8			
Presence of implants	No Yes	1 1.2 (0.6–2.3)	0.6			
CRP* at the first day of positive GN-BSI	< 100 mg/dl >=100 mg/dl	1 1.1 (0.6–2.2)	0.68			

*C-reactive protein

No risk factor was identified to explain an inadequate duration of antimicrobial therapy (Table S3).

## Discussion

To the best of our knowledge, this study is the first to compare the treatment approaches between cohorts of uGN-BSI and cGN-BSI, and to assess the risk factors linked to suboptimal treatment. Our results highlight an urgent need for improvement in both GN-BSI cohorts, particularly concerning the optimal timing of therapy escalation or de-escalation, which, in our study, occurred in only one-fourth of the patients. Additionally, the optimization of the transition to oral antibiotics remains critical, as it was infrequently (37.7%) and belatedly (after 6 days of treatment, IQR 4–8) implemented. Moreover, there is a necessity to refine the duration of antimicrobial therapy, which was on average 2 days longer than guideline recommendations suggest.

Only 24.7% of cases (48/194) in our study received optimal antimicrobial treatment within 24 h of antibiogram availability, irrespective of infection classification. This low rate of antibiotic de-escalation (ADE) persists despite the availability of ID/ABS specialists and rapid diagnostic susceptibility testing, both proven to improve outcomes and expedite treatment [5]. At our institution, microbiologists notify clinicians of every GN-BSI case on the day of blood culture positivity communicating gram stain or MALDI-ToF identification as well as results from rapid antimicrobial susceptibility testing (RAST) if applicable, enabling treatment escalation when needed (delayed or inadequate escalation despite antibiogram availability occurred in only 3.6% of cases). However, appropriate de-escalation post-antibiogram largely depends on the attending physician’s judgment and is often delayed until discharge or transfer. ID consultations were sought in only 12.9% (25/194) of cases, often late, especially in complicated GN-BSI, with a median delay of 6.5 days after the first positive blood culture (IQR 2.5–11.5 days). Delayed optimal treatment was more pronounced in cGN-BSI cases, with median delays of 4 days, sometimes up to 6 days. While cGN-BSI was not a significant risk factor for suboptimal treatment (OR 1.1, *p* = 0.76), clinicians often exercise caution in de-escalation, particularly for immunosuppressed patients, ICU cases, delayed clinical improvement, or unclear infection sources. ADE remains challenging, despite robust clinical support for its safety and efficacy [18]. High-quality evidence on its prognostic impact is limited, and no standardized ADE protocol exists, partly due to the heterogeneity of GN-BSI sources and pathogens [19]. Nevertheless, recent studies underscore ADE’s importance, even in severe infections [13]. A large retrospective study of 7,742 patients linked ADE with reduced resistance development [14], while others reported fewer adverse events and non-inferior outcomes [20–22]. However, integrating this knowledge into routine practice continues to present significant challenges.

Multivariate regression revealed that 3GCephRE isolates (OR 0.3, 95% CI 0.1–0.6, *p* < 0.01) and AmpC producers (OR 0.3, 95% CI 0.1–0.7, *p* < 0.01) were associated with the lowest risk of suboptimal treatment. Current guidelines recommend carbapenems for severe infections caused by AmpC producers in GN-BSI [23, 24]. Although cefepime is an alternative to carbapenems for AmpC infections, it was rarely used in our cohort due to its often classification as resistant under EUCAST expert rules [25] and the lower EUCAST breakpoint compared to CLSI [26]. For GN-BSI caused by ESBL-producing 3GCephRE isolates, carbapenems are similarly recommended as first-line therapy [24, 27]. So, this association of AmpC producers and 3GCephRE with optimal treatment should be interpreted cautiously because likely reflects the frequent use of empiric meropenem therapy, which often proved optimal without the need for antibiogram-guided adjustments. In contrast, uGN-BSI with source from urinary tract, which comprised the majority of GN-BSI cases, showed a higher risk of suboptimal treatment compared to other GN-BSI (univariate OR 2.1, *p* = 0.05, data not shown) though this was not significant in multivariate analysis. These cases, often involving wild-type *E. coli*, likely represent scenarios where AMS intervention to support de-escalation would be most beneficial.

Oralization was conducted in only 37.7% (63/167) of patients, and even among those with uGN-BSI, less than half received oral therapy (43%, 40/93). Furthermore, logistic regression identified uGN-BSI as having the strongest association with missed opportunities for oralization (cGN-BSI OR 0.5 (CI95% 0.2-1, compared to uGN-BSI). This likely reflects uGN-BSI patients’ greater suitability for oral therapy and lower resistance rates to recommended agents like TMP/SMX and ciprofloxacin. Additionally, clinicians often lacked awareness of evidence-based recommendations for early oral switches, with oralization in uGN-BSI cases occurring late (median 6 days, IQR 4–8). Several studies have shown that switching to oral substances after fewer days can be considered safe and offers potential benefits [11, 28, 29]. In this study, oralization often coincided with hospital discharge (median 7 days, IQR 5–11), leading to unnecessary treatment prolongation instead of improving outcomes like shorter hospital stays, reduced i.v. therapy complications, and lower costs.

Ciprofloxacin (44.4%, 28/63) and amoxicillin/clavulanate (27%, 17/63) were the most used oral agents, while TMP/SMX was used in only 7.9% (5/63). The choice of oral therapy in GN-BSI remains debated. A meta-analysis linked ß-lactams to higher recurrence rates [7], but another study on urinary-source GN-BSI found no significant difference compared to fluoroquinolones or TMP/SMX [30].

Current guidelines and ID experts now recommend shorter treatment durations for GN-BSI, with 7 days advised for uncomplicated cases, based on evidence demonstrating non-inferiority of shorter courses [8–10, 12, 15, 31]. In our study, the median treatment duration was 11 days (IQR 8–14), significantly longer for cGN-BSI. Contrary to expectations, no specific risk factors for prolonged therapy were identified. This may reflect a general lack of clinician awareness about the safety of shorter treatment courses, particularly for uGN-BSI. Notably, treatment duration for cases with an unknown source, potentially at greater risk of excessive therapy despite clinical improvement, was not included in the assessment for the risk factors.

This study has several limitations. First, as a retrospective observational study relying solely on electronic medical records, it is susceptible to biases, including information bias. Although we excluded patients with unclear data or confounding comorbidities, the results should be interpreted with caution. Additionally, this monocentric study, conducted in a large tertiary-care university hospital, had a low rate of ID consultations, possibly reflecting systemic underestimation of their benefits in GN-BSI [32, 33]. There is no uniform definition for uGN-BSI versus cGN-BSI in literature. While early clinical response, known source of infection and source control can be found in most definitions, opinions on other factors diverge. We applied the Delphi criteria proposed by Heli EL et al. [15]. However, a recent study criticized this definition, identifying nonfermentative gram-negative bacteria, difficult-to-treat resistance profiles, and pulmonary infection sources as independent risk factors for increased all-cause mortality in GN-BSI, supporting their exclusion from the definition of uGN-BSI [34].

Advancements in the management of GN-BSI infections need to be accompanied by studies evaluating their real-world applicability and identify potential barriers in daily clinical practice. In this study, we aimed to provide an accurate representation of current clinical practice in a large university hospital to help guiding the next steps for AMS interventions. Future research using pre-defined schemas for drug de-escalation is required to confirm the space proposed in this study for de-escalation in GN-BSI and its impact on patient outcomes [35]. Also, the use of predictive models to avoid unnecessary use of broad-spectrum antibiotics might become an important factor for AMS strategies [36, 37]. Additional tools, such as electronic alerts, might enhance the adherence to appropriate therapy durations [38]. 

In conclusion, this study highlights opportunities to improve de-escalation, oralization, and shorter treatment durations in GN-BSI. uGN-BSI are prime targets for AMS interventions, while cGN-BSI require tailored strategies and individualized approaches. Future research should focus on adapting current practices to better address the complexities of cGN-BSI, providing robust evidence to guide clinicians and support adherence to optimized treatment protocols.

## Supplementary Information

Below is the link to the electronic supplementary material.

Supplementary Material 1

## Data Availability

No datasets were generated or analysed during the current study.
