# Supplementary material for: Gram-negative bloodstream infections: where can we do better? A retrospective cohort study
Source: Eur J Clin Microbiol Infect Dis. 2025 Aug 9;44(11):2685–94. doi: 10.1007/s10096-025-05231-4 (PMC12619705; doi:10.1007/s10096-025-05231-4)
Supplement: Supplementary file 1 — Supplementary Material 1 [file 10096_2025_5231_MOESM1_ESM.docx]

**Gram-negative bloodstream infections: where can we do better? A retrospective cohort study. Supplementary Material**

Manouc Guit^1,2^, Konstantin Tanida^1,2^, Nicole Degel-Brossmann^1^, Martin Christner^1^, Martin Aepfelbacher^1^, Holger Rohde^1,2^, Flaminia Olearo^1,2^.

1. Center for Diagnostics, Institute of Medical Microbiology, Virology and Hygiene, University Medical Center Hamburg-Eppendorf, Hamburg, Germany.

2. Antimicrobial Stewardship Team, University Medical Center Hamburg-Eppendorf, Hamburg, Germany.

Corresponding author: Flaminia Olearo

Center for Diagnostics, Institute of Medical Microbiology, Virology and Hygiene, University Medical Center Hamburg-Eppendorf, Hamburg, Germany

Email : [f.olearo@uke.de](mailto:f.olearo@uke.de)

Telephone: +49 (0) 40 7410 - 53150

**Figure S1**. Kaplan-Meier plot showing time to optimal treatment, stratified by uncomplicated and complicated GN-BSI (p-value = 0.12).

**Figure S2.** Occasions of antimicrobial therapy optimization in terms of duration, oralization, de-escalation, escalation, and dosage, stratified by uncomplicated and complicated GN-BSI. Therapy Duration was suboptimal in 75 cases (38.7%, not applicable in 42 cases, 21.6%) overall, with 50 cases (49.5%, not applicable in 2 cases, 2.0%) in uncomplicated GN-BSI and 25 cases (26.9%, not applicable in 40 cases, 43,0%) in cGN-BSI. Oralisation could be optimized in 129 cases (66.5%) overall, 77 cases (76.2%) for uncomplicated GN-BSI, and 52 cases (55.9%) for cGN-BSI. De-escalation was optimizable in 140 cases (72.2%) overall, with 77 cases (76.2%) in uGN-BSI and 63 cases (67.7%) in cGN-BSI. Therapy Escalation should have occurred in 7 cases (3.6%) overall, 1 case (1.0%) for uGN-BSI and 6 cases (6.5%) for cGN-BSI. Dosing was incorrect in 3 cases (1.5%), all in uGN-BSI (3 cases, 3.0%). *not applicable: In 42 cases duration could not be assessed because of unknown focus, death, absence of source control or missing guideline information on therapy duration

**Table S1.** Comparison of antimicrobial therapy between complicated and uncomplicated GN-BSI.

|  | Overall GN-BSI (n=194) | uGN-BSI (n=101) | cGN-BSI (n=93) | Comparison between cGN-BSI and uGN-BSI  (p-value) |
| --- | --- | --- | --- | --- |
| Duration of antimicrobial therapy, median days (IQR)(range) | 11 (8-14)(4-105) | 10 (7-13) (4-32) | 12 (9-15)(5-105) | <0.01 |
| Difference between real and expected antimicrobial therapy duration, median (IQR)(range) | 2 (0-5)(-3 – 25) | 3 (0-5 ) (-2 - 25) | 2 (0 - 5)(-3 – 21) | 0.49 |
| Oralisation*, n (%) | 63 (37.7) | 40 (43) | 23 (31.1) | 0.11 |
| Time to oralisation, median (IQR)(range) | 6 (4-8)(1-29) | 5.5 (4-7) (2-11) | 6.5 (4-12)(1-29) | 0.12 |
| Antibiotics use for oralisation, n (%) * | Amox/Clav 17 (27)  Ciprofloxacin, 28 (44.4)  TMP/SMX, 5 (7.9)  Amoxicillin, 4 (6.4)  Cefpodoxim, 3 (4.7)  Cefalexin, 1 (1.6)  Levofloxacin, 2 (3.2)  Norfloxacin, 1 (1.6)  PIV ,1 (1.6)  Clindamycin, 1 (1.6) | Amox/Clav 12 (30)  Ciprofloxacin, 16 (40)  TMP/SMX 2 (5)  Amoxicillin 4 (10)  Cefpodoxim 3 (7.5)  Cefalexin 1 (2.5)  Levofloxacin 1 (2.5)  PIV 1 (2.5) | Amox/Clav 5 (21.7)  Ciprofloxacin 12 (52.1)  TMP/SMX 3 (13)  Levofloxacin 1 (4.4)  Norfloxacin 1 (4.4)  Clindamycin 1 (4.4) | _ |

**Table S2**. Risk factors for occasion lost for oralization in univariate and multivariate model (N=194)

|  |  | Univariate | P value | Multivariate | P value |
| --- | --- | --- | --- | --- | --- |
| Uncomplicated BSI  Complicated BSI |  | 1  0.4 (0.2-0.7) | <0.01 | 1  0.5 (0.2-1) | 0.07 |
| Age | <=64 y-o  >65 | 1  1.5 (0.8-2.8) | 0.14 | 1  1.1 (0.5-2) | 0.83 |
| Sex | Female  Male | 1  1 (0.6-1.9) | 0.87 | 1  1.3 (0.7-2.4) | 0.47 |
| Source | Urinary  Unknown  Abdominal  Pulmonary  Endovascular device  Wound  Other | 1  0.6 (0.2-1.6)  2.3 (1-5.2)  2.1 (0.5-9.2)  1.1 (0.3-4.1)  0.9 (0.2-4.8)  0.7 (0.1-5.6) | 0.27  0.05  0.32  0.86  0.93  0.73 | 1  0.8 (0.3-2.4)  2.1 (0.9-5)  2.4(0.5-11.1)  1.2 (0.3-4.6)  0.9 (0.2-5.1)  0.8 (0.1-6.8) | 0.68  0.08  0.24  0.78  0.96  0.28 |
| Bacteria | *E. Coli*  AmpC  *Klebsiella spp.*  *P. mirabilis*  *P. aeruginosa* | 1  0.7 (0.3-1.7)  0.9 ( 0.4-1.9)  5.2 ( 0.6-42)  1.4 (0.4-4.8) | 0.3  0.84  0.13  0.3 | _ |  |
| Third generation cephalosporin resistant (3GCephRE) | Not resistent  Resistent | 1  0.5 (0.2-1.2) | 0.12 | _ |  |
| Immunosuppression | No  Yes | 1  0.68 (0.3-1.3) | 0.26 | _ |  |
| Charlson Comorbidity Index (CCI) | >5  3-4  1-2  0 | 1  0.8 ( 0.4-1.6)  0.6 (0.3-1.5)  0.6 (0.2-1.7) | 0.47  0.26  0.34 | _ |  |
| Presence of implants | No  Yes | 1  1.2 | 0.5 | _ |  |
| CRP at the first day of positive GN-BSI | <100 mg/dl  >=100 mg/dl | 1  1.3 (0.7-2.3) | 0.43 | _ |  |

**Table S3**. Risk factors for inadequate duration of antimicrobial treatment in univariate and multivariate model (N=194)

|  |  | Univariate | Pvalue | Multivariate | P value |
| --- | --- | --- | --- | --- | --- |
| Uncomplicated BSI  Complicated BSI |  | 1  0.8 (0.4-1.7) | 0.69 | 1  0.9 (0.44-1.8) | 0.77 |
| Age | <=64 y-o  >65 | 1  1.1 (0.6-2.1) | 0.73 | 1  1 (0.5-2) | 0.88 |
| Sex | Female  Male | 1  0.8 (0.5-1.6) | 0.62 | 1  0.8 (0.5-1.6) | 0.65 |
| Bacteria | *E. Coli*  AmpC  Klebsiella spp.  *P. mirabilis*  *P. aeruginosa* | 1  1.4(0.5-4.2)  0.8 ( 0.3-1.7)  2.1 ( 0.7-11.9)  0.9 (0.3-5.5) | 0.52  0.53  0.41  0.65 | _ |  |
| Third generation cephalosporin resistant | Not resistent  Resistent | 1  0.97 (0.7-1.3) | 0.87 | _ |  |
| Immunosuppression | No  Yes | 1  1 (0.5-2.3) | 0.93 | _ |  |
| Charlson index | >5  3-4  1-2  0 | 1  0.9 ( 0.4-2.1)  1.5 (0.3-3.7)  1.3 (0.4-4.2) | 0.87  0.36  0.67 | _ |  |
| Presence of implants | No  Yes | 1  0.6(0.3-1.2) | 0.19 | _ |  |
| CRP at the first day of positive GN-BSI | <100 mg/dl  >=100 mg/dl | 1  1.6 (0.8-3.1) | 0.16 | _ |  |
